# Liver Function—How to Screen and to Diagnose: Insights from Personal Experiences, Controlled Clinical Studies and Future Perspectives

**DOI:** 10.3390/jpm12101657

**Published:** 2022-10-05

**Authors:** Matthias Buechter, Guido Gerken

**Affiliations:** 1 Department of Gastroenterology and Hepatology, Elisabeth Hospital, 58638 Iserlohn, Germany; 2 Department of Gastroenterology and Hepatology, University Hospital Essen, University of Duisburg-Essen, 45147 Essen, Germany

**Keywords:** liver disease, liver function, LiMAx, elastography

## Abstract

Acute and chronic liver disease is a relevant problem worldwide. Liver function plays a crucial role in the course of liver diseases not only in estimating prognosis but also with regard to therapeutic interventions. Within this review, we discuss and evaluate different tools from screening to diagnosis and give insights from personal experiences, controlled clinical studies and future perspectives. Finally, we offer our novel diagnostic algorithm to screen patients with presumptive acute or chronic liver disease in the daily clinical routine.

Acute and chronic liver diseases are major causes of morbidity and mortality worldwide. Nowadays, liver cirrhosis is the most common non-neoplastic cause of death in Europe and the U.S. among diseases of the gastrointestinal tract [1,2,3]. From a pathophysiological point of view, acute or chronic inflammation leads to destruction and progressive fibrosis of the liver parenchyma. Liver cirrhosis represents the endpoint of this frequently creeping silent process lasting for years or even decades [4]. Ongoing liver damage with an increase in portal pressure leads to the development of portal hypertension (PH), which is a crucial factor in the history of cirrhosis. When portal-hypertension-induced hepatic decompensation events such as ascites, variceal bleeding, hepatic encephalopathy or hepatocellular carcinoma occur, mortality increases rapidly [5,6,7]. Acute-on-chronic liver failure (ACLF), defined as acute decompensation of chronic liver disease associated with (multiple) organ failures, is probably the most serious acute decompensation of cirrhosis with reported mortality rates of up to 80%, despite new insights in pathophysiology and optimal treatment [8,9,10].

Fortunately, progress in diagnostics and therapy over the last decades has improved the prognosis of patients suffering from chronic liver disease (CLD). Nowadays, a variety of different treatment modalities have become available depending upon the etiology and stage of CLD. However, the management of patients with CLD becomes more and more challenging since there is a clear focus on individualized medicine taking into account not only the patient’s underlying liver disease and its severity but also co-morbidities and disease-modifying factors.

When establishing treatment strategies for primary hepatocellular carcinoma (HCC), for example, we have to include important pathogenetic aspects into our personalized decision-making process. On the one hand, there is the tumor burden of the patient. On the other hand, there is the host with all individual co-morbidities and risk factors modifying the course and progression of the tumor disease. In addition, the molecular and immunological interaction between host and tumor is very important. Finally, different local and systemic targeted HCC treatment options have to be evaluated carefully not only regarding their effect on the tumor itself but also on their influence to downregulate liver function and liver capacity [11,12,13].

However, the liver itself is one of the most important human solid organs with multiple functions. Thus, liver cells cover not only metabolic homeostasis but also metabolism, production of nutrients such as carbohydrates, fats, proteins, vitamins, distribution, storage and synthesis of proteins and final metabolic products including drugs and toxins invading the liver cell cycle.

The first and important step in the evaluation of liver function is the complete and proper clinical examination of the patient. Patients with liver cirrhosis may have numerous physical examination findings that reflect the severity of the underlying liver disease. Although some symptoms and signs related to advanced CLD are nonspecific (e.g., abdominal pain, nausea, malaise), some findings are more characteristic and indicate complications of liver disease [14]. Key physical findings in patients with cirrhosis are, for example, hepatomegaly, splenomegaly, dilated abdominal veins, spider nevi and palmar erythema, whereas ascites, gastrointestinal bleeding, jaundice and encephalopathy point to hepatic decompensation and advanced portal hypertension with significantly impaired liver function [6,15,16]. In addition, severe muscle depletion (sarcopenia) resulting from an imbalance between protein synthesis and breakdown in patients with advanced stages of CLD is related to increased complications [17,18,19]. Moreover, a recently published study showed that body fat composition determines outcomes in patients with cirrhosis, while low subcutaneous fat levels were associated with a higher rate of cirrhosis-associated complications and mortality [20]. 

The next practical clinical approach to determine the stage and severity of liver disease covers different diagnostic tools using conventional blood parameters or so-called “serum biomarkers” such as cellular enzymes, albumin, bilirubin and coagulation proteins, as well as simple scoring systems based on these parameters. 

Then, different imaging techniques are usually applied to visualize the hepatic parenchyma, its surface and perfusion. Essentially, ultrasound is the first-line approach which is often combined with a high-resolution computed tomography (CT) scan and/or magnetic resonance imaging (MRI). CT scans and MRI play an important role in the objective assessment of cirrhosis and its complications (e.g., focal liver lesions, HCC, tumor staging, portal vein thrombosis, ascites). However, due to its limited availability (MRI), radiation exposure (CT scan) and poor diagnostic accuracy in less advanced stages of fibrosis, these techniques are not well suited as screening or monitoring tools in chronic liver disease [21,22,23]. 

Finally, immunological, biological and genetic markers should be taken into consideration. Although non-invasive techniques have been extensively examined with good evidence, liver histology obtained either by percutaneous or mini-laparoscopy-guided biopsy still plays an important role in the assessment of acute or chronic liver diseases, especially at first diagnosis and in uncertain situations [24,25,26]. Still, this invasive approach offers the unique possibility of direct tissue analysis with different techniques such as immunohistochemistry and molecular pathology. However, due to its invasiveness and risk of complications (especially bleeding), liver biopsy is unsuitable for screening and monitoring patients suffering from CLD, particularly when repeated interventions are required [27,28]. Furthermore, the gained histological specimen only represents 1/50,000 of the whole liver tissue and might therefore not reflect the true degree of inflammation, fibrosis or cirrhosis, despite an adequate sample size in diffuse parenchymal liver diseases. In addition to the problem of “intraobserver variation”, this so-called “sampling error” may lead to over- or underestimation of the real extent and severity of CLD with relevant consequences [29,30,31].

The enzymatic machinery in liver cell cytoplasm plays an important role in the evaluation of liver disease as it represents the basis for determination of liver function containing, on one hand, parameters of cholestasis, e.g., bilirubin, alkaline phosphatase and gamma-glutamyltransferase, and on the other hand, transaminases as inflammation markers, e.g., aspartate aminotransferase (AST), alanine aminotransferase (ALT) and glutamate dehydrogenase (GLDH), as well as synthesis parameters such as albumin, cholinesterase and coagulation factors, e.g., factors I, II, V, VII, VIII and X, respectively. Determination of liver transaminases (e.g., AST, ALT) is easy to perform, and they are therefore well suited as screening parameters. Although temporary elevation of these enzymes is not a harmful problem, higher and repeatedly elevated levels directly correlate with increased mortality [32,33,34]. Hence, it is important that family doctors focus on that. Usually, an increase in ALT levels is compatible with hepatocellular damage (“hepatocellular pattern”), while an increase in cholestasis markers such as alkaline phosphatase represents a cholestatic liver disease (“cholestatic pattern”) [35]. A mixed pattern of these markers can serve as a hint to diseases with both aspects. In addition, determination of the exact levels of serum transaminases can help to classify the activity, severity and stage of the present liver disease. For example, acute and fulminant viral hepatitis is accompanied by very high transaminase levels, whereas chronic active liver disease comes along with mild elevation of these enzymes. Interestingly, in patients with advanced stages of liver disease including liver cirrhosis, liver enzymes are not infrequently only slightly elevated or even within normal ranges. Overall, none of these enzymes or molecules summarized under the term “serum biomarkers” are of use by themselves but are useful when combined with each other or certain clinical parameters in marker panels or mathematical scoring systems in order to assess the extent of CLD. The so-called “non-invasive fibrosis scores” such as the AST-to-ALT ratio (AAR), AST-to-platelet ratio index (APRI), fibrotest, NAFLD fibrosis score (NFS) or fibrosis-4 (FIB-4) score have been established in recent years for clinical use and can help to indirectly quantify the stage of liver fibrosis [21,36,37,38]. Besides the advantage of non-invasiveness, these scores are ubiquitously available and cost-effective. However, their prognostic value is not clear-cut, since they are useful in the exclusion of advanced fibrosis and cirrhosis (sensitivity 64-92%, specificity 38-75%, AUROC 0.74-0.88) but do not distinguish well early and intermediate stages of fibrosis [21,38,39,40,41,42,43]. In addition, the Child–Pugh Score (CPS), the Albumin–Bilirubin (ALBI) Score and the Model of End-Stage Liver Disease (MELD) Score can be used to evaluate actual liver function and estimate prognosis [44,45,46,47]. However, all these scoring systems have to be interpreted with caution since they include subjective evaluation (e.g., extent of ascites for the CPS) and harbor several pitfalls such as inflammation and malnutrition, which can influence relevant parameters such as albumin as an integral part of those scores. Furthermore, various laboratory data are not liver-specific (e.g., AST elevation due to alcohol abuse or muscle breakdown) and are biased by various factors such as deficiencies, (iatrogenic) substitution, drugs (e.g., downregulation of clotting factors by vitamin K antagonists), extrahepatic causes of, for example, thrombocytopenia or biological half-life of these enzymes. 

Conventional abdominal ultrasound (US) is unequivocally the most common and widely used imaging modality among patients with presumptive or established acute or chronic liver disease. This technique offers the opportunity to visualize hepatic parenchyma, its morphology and perfusion when combined with Doppler sonography. Characteristic findings of liver cirrhosis in US are nodular liver surface, round edge, inhomogeneous parenchyma with hypoechoic nodules, hypertrophy of the caudate segment and rarefication of liver veins [48,49]. The diagnostic accuracy in individuals with advanced cirrhosis is high (sensitivity and specificity > 90%), particularly when signs of (decompensated) portal hypertension (e.g., ascites, varices, splenomegaly) are present [50,51]. However, the accuracy of US in diagnosing fibrosis or (beginning) cirrhosis in the absence of portal hypertension is significantly inferior since liver morphology may be normal in these stages [21,52,53,54]. Although abdominal US is standardized in many countries (e.g., DEGUM certification in Germany), the quality of the examination highly depends on the skill, knowledge and experience of the investigator [55,56]. However, abdominal US does not only play a role in the initial diagnosis but is also very important in the surveillance of patients suffering from CLD: it is recommended as an integral part in the follow-up of cirrhotic patients in multiple international guidelines. In this regard, its fundamental importance lies in the early detection of complications such as the development of ascites, portal vein thrombosis or hepatocellular carcinoma (HCC), which strongly impacts patient management, outcome and economic burden of healthcare systems [57,58,59]. If a focal hepatic lesion in cirrhosis is detected by US, its characterization can be performed by injection of a contrast medium (contrast-enhanced ultrasound; CEUS). However, the role of CEUS in the detection and characterization of focal hepatic lesions is still discussed controversially, and its relevance is weighted differently in international guidelines. Nowadays, the combination of CEUS with high-resolution CT/MRI and determination of alpha-fetoprotein (AFP) is adequate to diagnose HCC (without the necessity of tissue acquisition by biopsy) [11,13,60,61,62]. 

During the last decades, ultrasound-based elastography has been introduced as a novel non-invasive imaging modality to stage liver fibrosis. By the use of transient elastography (TE) or shear-wave elastography (SWE) the stiffness (or hardness) of the liver tissue can be measured by low-frequency vibrations, which is supposed to be proportional to the extent of fibrosis [63,64,65,66]. Since progressive scarring of the liver parenchyma during the course of CLD leads to increasing elasticity, this technique can therefore not only aid in the initial characterization of disease severity but also serve as a tool for monitoring and treatment follow-up. Besides the advantage of non-invasivity, elastography is easy to perform, cost-effective and highly reproducible. There is reliable scientific evidence for this method strengthened by numerous studies, especially in the early and late stages of fibrosis (specificity and sensitivity > 90%) [67,68,69,70]. In advanced cirrhosis, liver and spleen elastography can provide additional information about the presence and extent of portal hypertension (PH), since they are positively correlated with the hepatic venous pressure gradient (HVPG) and decompensation events such as variceal bleeding [71,72]. However, anatomic conditions (e.g., body fat, presence of ascites, short rib distance) reduce its feasibility in approximately 10% of patients in whom valuable results cannot be attained. Furthermore, the results can be invalid or distorted by steatosis, congestion and inflammatory activity, particularly in terms of concomitant acute liver damage [36,73]. 

Decision making in clinical hepatology often requires the assessment of liver function. However, all the above-mentioned parameters are indirect markers for liver function and acute or chronic liver damage and therefore somehow more or less lack precision. Thus, it is sometimes difficult to assess the liver’s response to various insults, formulate a treatment approach and predict recovery by the use of these techniques [74]. However, how can we nowadays assess actual metabolic liver function more precisely?

Enzymatic liver function tests have been employed experimentally and clinically for several decades. What these tests have in common is that a metabolite of a usually intravenously applied substrate is measured in blood samples or exhaled air. The perfect substrate should be metabolized only in hepatocytes and therefore selectively reflect metabolic liver function. Different substrates of the cytochrome P-450 system such as ^13^C-aminopyrine, ^13^C-phenylalanine, ^13^C-galactose, ^13^C-methionine or monoethylglycinexylidide (MEGX) have been introduced and further investigated to estimate liver function during the last decades [74,75,76,77,78,79]. Moreover, indocyanine green (ICG) has been used to quantitatively assess liver function and hepatic clearance [80,81,82]. However, due to different reasons, these tests have not been established sustainably in the clinical routine. 

More recently, the dynamic measurement of the enzymatic liver function by the liver maximum capacity (LiMAx) breath test has been introduced as a robust technique to determine dynamic liver function based on the specific hepatic cytochrome p 450 1A 2 metabolism of an intravenously injected substance [83]. After application of the substrate _13_C methacetin, this is immediately demethylated into acetaminophen and _13_CO_2_ in hepatocytes. Then, the concentration of _13_CO_2_ is measured in exhalation, and the liver capacity can be calculated from the analysis of the _13_CO_2_/_12_CO_2_ ratio in relation to the individual baseline ratio prior to the substrate injection [84]. Thus, the procedure is visualized as a curve on the screen of a monitor. In healthy individuals, we usually can observe two parts of metabolic liver function. At first, there is a quickly rising curve representing the initial phase of the metabolizing process. After rapidly reaching the plateau (>500 μg/kg/h), usually after about 5 min, we see the long-term line representing the maintenance phase of liver function. If the liver function is significantly impaired (e.g., acute liver failure, advanced stages of cirrhosis), we observe completely different curve kinetics. After injection of the substrate, there is only one phase with a linear and very slowly rising gradient reaching the maximum not until the end of the examination after 60 min. In accordance, the maximum value is notably lower within this stadium (<100 μg/kg/h). On the other hand, in individuals with moderately impaired liver function (e.g., acute hepatitis, mild fibrosis), we see a sort of mixture of both curves. At first, there is a fast-rising curve similar to the healthy liver, although the gradient is less pronounced. After about 5 min, the gradient decreases to a slowly rising linear curve similar to the significantly impaired liver function but obviously being on a higher level. The three different curve kinetics of the LiMAx test are exemplarily demonstrated in Figure 1. In particular, we can therefore not only determine actual enzymatic function on the basis of the absolute and calculated liver maximum capacity value but also visualize, characterize and estimate dynamic hepatic reserve by the curve progression. 

We could confirm a good correlation between structural and functional changes in a cohort of 102 patients, in whom enzymatic liver function measured by LiMAx was closely associated with histologically proven parenchymal changes (fibrosis) and elastography determined by TE (Figure 2). However, the highest diagnostic accuracy of non-invasively detecting cirrhosis was reached by combining TE and LiMAx [85]. In addition, we found a strong correlation between different clinical stages occurring in the course of CLD and liver function in a great cohort of patients (n = 464) with CLD. Herein, the LiMAx test was even superior to TE, CPS, MELD and serum biomarkers with a Spearman’s correlation coefficient of −0.81 [86].

The LiMAx test offers the unique opportunity that alterations in liver function can be determined immediately and in real time. We here give an example of a young male patient with acute liver failure (ALF) of unknown origin with significantly impaired liver function complicated by sepsis, in whom the LiMAx test was the first parameter predicting beneficial outcome while laboratory parameters improved much later [87]. In particular, serial LiMAx measurements offer valuable additional information in the course of acute liver injury. We saw similar clinical courses in, for example, patients with ALF due to autoimmune hepatitis, where LiMAx was the first parameter estimating prognosis after initiation of high-dose steroids. These data were confirmed by two pilot studies in which LiMAx improved outcome prediction in ALF [88,89]. Likewise, estimating prognosis in CLD/cirrhosis is highly accurate by LiMAx and comparable to validated scoring systems such as the MELD [86,90]. The LiMAx test is therefore the first functional capacity test with added benefit to the current “standard of diagnostic care”. The findings of our group were confirmed by other tertiary European centers using the LiMAx test in Germany and the Netherlands [91,92,93,94,95].

Interventions in patients with CLD/liver cirrhosis may be associated with different complications. Further impairment of liver function, which can even lead to subsequent liver failure, is definitely the most important of them. Therefore, an adequate selection of patients suitable for a certain intervention is of fundamental importance. The LiMAx test was able to estimate prognosis with regard to liver surgery, transjugular intrahepatic portosystemic shunt (TIPS) or liver cancer therapies such as transarterial chemotherapy (TACE) or selective intra-arterial radiotherapy (SIRT; unpublished data) and decline from patients who will not benefit from these procedures [83,96,97,98,99,100,101]. However, it is not only possible to individually decide whether a certain intervention is possible but also to determine an appropriate timepoint. Chemotherapy-associated liver injury is a well-known phenomenon, and pre-operative chemotherapy is a major risk factor for postoperative liver failure. Herein, a study showed that LiMAx impairment was dependent on chemotherapy cycles and therapy-free intervals in patients receiving platin-based chemotherapy due to colorectal liver metastases. However, patients with an impaired LiMAx showed sufficient regeneration during chemotherapy cessation when surgery was postponed. Preoperative performance of the LiMAx test can therefore augment surgical strategy and timing of surgery after previous chemotherapy [102,103,104,105].

Artificial intelligence (AI) has come into the spotlight in medicine and gastroenterology. Hepatology is no exception, with a growing number of studies published that apply AI techniques to the diagnosis and treatment of liver diseases [106]. Deep learning models make it possible to extract clinically relevant information from diverse and complex clinical datasets. Imaging, laboratory data and histopathology, for example, contain information for detecting liver fibrosis, differentiating focal liver lesions and predicting the prognosis of chronic liver disease which AI can extract [107]. Ultimately, AI systems could be implemented in clinical routines as decision support tools [108].

To conclude, we have a bouquet of different diagnostic and prognostic tools to screen and estimate liver function in patients with acute and chronic liver diseases. These entities should be applied carefully and thoughtfully to our patients. With the LiMAx test, we have a strong and robust additional assay that offers the unique opportunity of specific, semiquantitative and dynamic measurement of actual enzymatic liver function, which we included in our novel diagnostic algorithm to screen patients with presumptive acute or chronic liver disease (Figure 3).

## Figures and Tables

**Figure 1:**
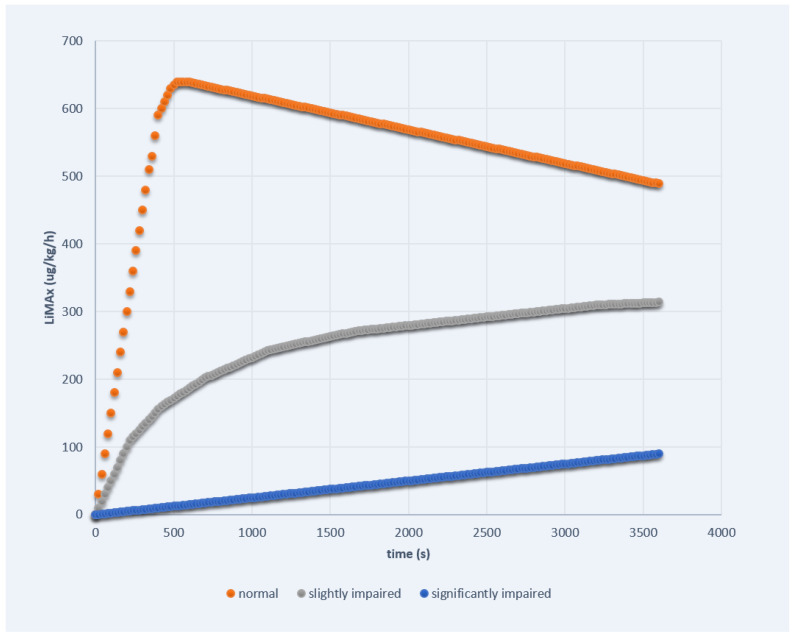
The three different curve kinetics of the LiMAx test.

**Figure 2:**
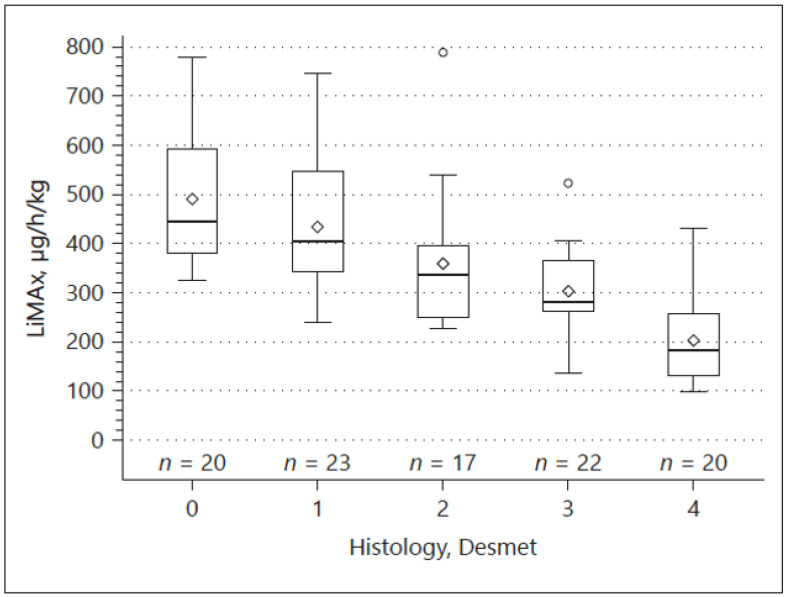
Correlation between LiMAx and histological specimen classified according to the Desmet scoring system.

**Figure 3:**
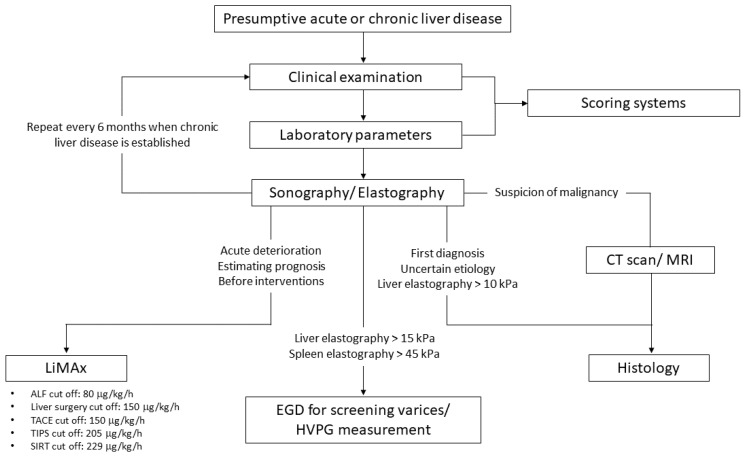
Proposed algorithm in individuals with presumptive acute or chronic liver disease.

## Data Availability

Data sharing not applicable.

## Abbreviations

AAR	AST-to-ALT ratio
ACLF	acute-on-chronic liver failure
AFP	alpha-fetoprotein
AI	artificial intelligence
ALBI Score	Albumin–Bilirubin Score
ALF	acute liver failure
ALT	alanine aminotransferase
APRI	AST-to-platelet ratio index
AST	aspartate aminotransferase
CEUS	contrast-enhanced ultrasound
CLD	chronic liver disease
CPS	Child–Pugh Score
CT	computed tomography
FIB-4 score	fibrosis-4 score
GLDH	glutamate dehydrogenase
HCC	hepatocellular carcinoma
HVPG	hepatic venous pressure gradient
LiMAx test	liver maximum capacity test
MELD Score	Model of End-Stage Liver Disease Score
MRI	magnetic resonance imaging
NFS	NAFLD fibrosis score
PH	portal hypertension
SIRT	selective intra-arterial radiotherapy
SWE	shear-wave elastography
TACE	transarterial chemotherapy
TE	transient elastography
TIPS	transjugular intrahepatic portosystemic shunt
US	ultrasound

## References

[1] Moon A.M., Singal A.G., Tapper E.B. (2020). Contemporary Epidemiology of Chronic Liver Disease and Cirrhosis. Clin. Gastroenterol. Hepatol..

[2] Bellentani S., Tiribelli C., Saccoccio G., Sodde M., Fratti N., de Martin C., Cristianini G. (1994). Prevalence of chronic liver disease in the general population of northern Italy: The dionysos study. Hepatology.

[3] Orman E.S., Roberts A., Ghabril M., Nephew L., Desai A.P., Patidar K., Chalasani N. (2019). Trends in Characteristics, Mortality, and Other Outcomes of Patients with Newly Diagnosed Cirrhosis. JAMA Netw. Open.

[4] Lee Y.A., Wallace M.C., Friedman S.L. (2015). Pathobiology of liver fibrosis: A translational success story. Gut.

[5] D’Amico G., Garcia-Tsao G., Pagliaro L. (2006). Natural history and prognostic indicators of survival in cirrhosis: A systematic review of 118 studies. J. Hepatol..

[6] Tsochatzis E.A., Bosch J., Burroughs A.K. (2014). Liver cirrhosis. Lancet.

[7] Iwakiri Y., Trebicka J. (2021). Portal hypertension in cirrhosis: Pathophysiological mechanisms and therapy. JHEP Rep..

[8] Belli L.S., Duvoux C., Artzner T., Bernal W., Conti S., Cortesi P.A., Sacleux S.-C., Pageaux G.-P., Radenne S., Trebicka J. (2021). Liver transplantation for patients with acute-on-chronic liver failure (ACLF) in Europe: Results of the ELITA/EF-CLIF collaborative study (ECLIS). J. Hepatol..

[9] Hernaez R., Solà E., Moreau R., Ginès P. (2017). Acute-on-chronic liver failure: An update. Gut.

[10] Moreau R., Jalan R., Gines P., Pavesi M., Angeli P., Cordoba J., Durand F., Gustot T., Saliba F., Domenicali M. (2013). Acute-on-Chronic Liver Failure Is a Distinct Syndrome That Develops in Patients with Acute Decompensation of Cirrhosis. Gastroenterology.

[11] Greten T.F., Malek N.P., Schmidt S., Arends J., Bartenstein P., Bechstein W., Bernatik T., Bitzer M., Chavan A., Dollinger M. (2013). Diagnosis of and therapy for hepatocellular carcinoma. Z. Gastroenterol..

[12] Couri T., Pillai A. (2019). Goals and targets for personalized therapy for HCC. Hepatol. Int..

[13] Su G.L., Altayar O., O’Shea R., Shah R., Estfan B., Wenzell C., Sultan S., Falck-Ytter Y. (2022). AGA Clinical Practice Guideline on Systemic Therapy for Hepatocellular Carcinoma. Gastroenterology.

[14] Wilson R., Williams D.M. (2022). Cirrhosis. Med. Clin. N. Am..

[15] Ginès M.D., Aleksander K., Juan G.A., Elsa S., Núria F., Patrick S.K. (2021). Liver cirrhosis. Lancet.

[16] Turco L., Garcia-Tsao G. (2019). Portal Hypertension: Pathogenesis and Diagnosis. Clin. Liver Dis..

[17] Ebadi M., Bhanji R.A., Mazurak V.C., Montano-Loza A.J. (2019). Sarcopenia in cirrhosis: From pathogenesis to interventions. J. Gastroenterol..

[18] Tandon P., Montano-Loza A.J., Lai J.C., Dasarathy S., Merli M. (2021). Sarcopenia and frailty in decompensated cirrhosis. J. Hepatol..

[19] Marasco G., Dajti E., Ravaioli F., Brocchi S., Rossini B., Alemanni L.V., Peta G., Bartalena L., Golfieri R., Festi D. (2020). Clinical impact of sarcopenia assessment in patients with liver cirrhosis. Expert Rev. Gastroenterol. Hepatol..

[20] Engelmann C., Aehling N.F., Schob S., Nonnenmacher I., Handmann L., Macnaughtan J., Herber A., Surov A., Kaiser T., Denecke T. (2022). Body fat composition determines outcomes before and after liver transplantation in patients with cirrhosis. Hepatol. Commun..

[21] Lurie Y., Webb M., Cytter-Kuint R., Shteingart S., Lederkremer G. (2015). Non-invasive diagnosis of liver fibrosis and cirrhosis. World J. Gastroenterol..

[22] Faria S.C., Ganesan K., Mwangi I., Shiehmorteza M., Viamonte B., Mazhar S., Peterson M., Kono Y., Santillan C., Casola G. (2009). MR Imaging of Liver Fibrosis: Current State of the Art. RadioGraphics.

[23] Hussain S.M., Reinhold C., Mitchell D.G. (2009). Cirrhosis and Lesion Characterization at MR Imaging. RadioGraphics.

[24] Rockey D.C., Caldwell S.H., Goodman Z.D., Nelson R.C., Smith A.D. (2009). Liver biopsy. Hepatology.

[25] Racanelli V., Rehermann B. (2006). The liver as an immunological organ. Hepatology.

[26] European Association for the Study of the Liver (EASL), European Association for the Study of Diabetes (EASD), European Association for the Study of Obesity (EASO) (2016). EASL-EASD-EASO Clinical Practice Guidelines for the management of non-alcoholic fatty liver disease. J. Hepatol..

[27] Atwell T.D., Smith R.L., Hesley G.K., Callstrom M.R., Schleck C.D., Harmsen W.S., Charboneau J.W., Welch T.J. (2010). Incidence of Bleeding After 15,181 Percutaneous Biopsies and the Role of Aspirin. AJR Am. J. Roentgenol..

[28] Tian G., Kong D., Jiang T., Li L. (2020). Complications after Percutaneous Ultrasound-Guided Liver Biopsy: A Systematic Review and Meta-analysis of a Population of More Than 12,000 Patients from 51 Cohort Studies. J. Ultrasound. Med..

[29] Sumida Y., Nakajima A., Itoh Y. (2014). Limitations of liver biopsy and non-invasive diagnostic tests for the diagnosis of nonalcoholic fatty liver disease/nonalcoholic steatohepatitis. World J. Gastroenterol..

[30] Goldstein N.S., Hastah F., Galan M.V., Gordon S.C. (2005). Fibrosis heterogeneity in nonalcoholic steatohepatitis and hepatitis C virus needle core biopsy specimens. Am. J. Clin. Pathol..

[31] Regev A., Berho M., Jeffers L.J., Milikowski C., Molina E.G., Pyrsopoulos N.T., Feng Z., Reddy K.R., Schiff E.R. (2002). Sampling error and intraobserver variation in liver biopsy in patients with chronic HCV infection. Am. J. Gastroenterol..

[32] Unalp-Arida A., Ruhl C.E. (2016). Noninvasive fatty liver markers predict liver disease mortality in the U.S. population. Hepatology.

[33] Kim H.C., Nam C.M., Jee S.H., Han K.H., Oh D.K., Suh I. (2004). Normal serum aminotransferase concentration and risk of mortality from liver diseases: Prospective cohort study. BMJ.

[34] Ruhl C.E., Everhart J.E. (2009). Elevated serum alanine aminotransferase and gamma-glutamyltransferase and mortality in the United States population. Gastroenterology.

[35] Vagvala S.H., O’Connor S.D. (2018). Imaging of abnormal liver function tests. Clin. Liver Dis..

[36] Loomba R., Adams L.A. (2020). Advances in non-invasive assessment of hepatic fibrosis. Gut.

[37] Vilar-Gomez E., Chalasani N. (2018). Non-invasive assessment of non-alcoholic fatty liver disease: Clinical prediction rules and blood-based biomarkers. J. Hepatol..

[38] Angulo P., Hui J.M., Marchesini G., Bugianesi E., George J., Farrell G.C., Enders F., Saksena S., Burt A.D., Bida J.P. (2007). The NAFLD fibrosis score: A noninvasive system that identifies liver fibrosis in patients with NAFLD. Hepatology.

[39] Ding D., Li H., Liu P., Chen L., Kang J., Zhang Y., Ma D., Chen Y., Luo J., Meng Z. (2015). FibroScan, aspartate aminotransferase and alanine aminotransferase ratio (AAR), aspartate aminotransferase to platelet ratio index (APRI), fibrosis index based on the 4 factor (FIB-4), and their combinations in the assessment of liver fibrosis in patients with hepatitis B. Int. J. Clin. Exp. Med..

[40] Yuan X., Duan S.-Z., Cao J., Gao N., Xu J., Zhang L. (2019). Noninvasive inflammatory markers for assessing liver fibrosis stage in autoimmune hepatitis patients. Eur. J. Gastroenterol. Hepatol..

[41] European Association for Study of Liver, Asociacion Latinoamericana para el Estudio del Higado (2015). EASL-ALEH Clinical Practice Guidelines: Non-invasive tests for evaluation of liver disease severity and prognosis. J. Hepatol..

[42] Petta S., Wong V.W.-S., Cammà C., Hiriart J.-B., Wong G.L.-H., Vergniol J., Chan A., Di Marco V., Merrouche W., Chan H.L.-Y. (2017). Serial combination of non-invasive tools improves the diagnostic accuracy of severe liver fibrosis in patients with NAFLD. Aliment. Pharmacol. Ther..

[43] Castera L., Pinzani M. (2010). Biopsy and non-invasive methods for the diagnosis of liver fibrosis: Does it take two to tango?. Gut.

[44] Ruf A., Dirchwolf M., Freeman R.B. (2022). From Child-Pugh to MELD score and beyond: Taking a walk down memory lane. Ann. Hepatol..

[45] Papatheodoridis G.V., Cholongitas E., Dimitriadou E., Touloumi G., Sevastianos V., Archimandritis A.J. (2005). MELD vs Child-Pugh and creatinine-modified Child-Pugh score for predicting survival in patients with decompensated cirrhosis. World J. Gastroenterol..

[46] Shao L., Han B., An S., Ma J., Guo X., Romeiro F.G., Mancuso A., Qi X. (2017). Albumin-to-bilirubin score for assessing the in-hospital death in cirrhosis. Transl. Gastroenterol. Hepatol..

[47] Wang J., Zhang Z., Yan X., Li M., Xia J., Liu Y., Chen Y., Jia B., Zhu L., Zhu C. (2019). Albumin-Bilirubin (ALBI) as an accurate and simple prognostic score for chronic hepatitis B-related liver cirrhosis. Dig. Liver Dis..

[48] Matsutani S., Fukuzawa T., Ebara M., Ohto M. (1994). Ultrasonography in the diagnosis of liver cirrhosis. Nihon Rinsho.

[49] Tchelepi H., Ralls P.W., Radin R., Grant E. (2002). Sonography of diffuse liver disease. J. Ultrasound. Med..

[50] Kelly E.M.M., Feldstein V.A., Parks M., Hudock R., Etheridge D., Peters M.G. (2018). An Assessment of the Clinical Accuracy of Ultrasound in Diagnosing Cirrhosis in the Absence of Portal Hypertension. Gastroenterol. Hepatol..

[51] Yen Y.-H., Kuo F.-Y., Chen C.-H., Hu T.-H., Lu S.-N., Wang J.-H., Hung C.-H. (2019). Ultrasound is highly specific in diagnosing compensated cirrhosis in chronic hepatitis C patients in real world clinical practice. Medicine.

[52] Allan R., Thoirs K., Phillips M. (2010). Accuracy of ultrasound to identify chronic liver disease. World J. Gastroenterol..

[53] Colli A., Fraquelli M., Andreoletti M., Marino B., Zuccoli E., Conte D. (2003). Severe Liver Fibrosis or Cirrhosis: Accuracy of US for Detection—Analysis of 300 Cases. Radiology.

[54] Ong T.Z., Tan H.J. (2003). Ultrasonography is not reliable in diagnosing liver cirrhosis in clinical practice. Singap. Med. J..

[55] Welle R., Seufferlein T., Kratzer W. (2021). Current state of under- and postgraduate education in abdominal ultrasonography at German university hospitals. A panel study over 20 years. Z. Gastroenterol..

[56] Maruyama H., Kato N. (2019). Advances in ultrasound diagnosis in chronic liver diseases. Clin. Mol. Hepatol..

[57] European Association for the Study of the Liver (2018). EASL Clinical Practice Guidelines for the management of patients with decompensated cirrhosis. J. Hepatol..

[58] Gerbes A.L., Labenz J., Appenrodt B., Dollinger M., Gundling F., Gülberg V., Holstege A., Lynen-Jansen P., Steib C.J., Trebicka J. (2019). Updated S2k-Guideline “Complications of liver cirrhosis”. German Society of Gastroenterology (DGVS). Z. Gastroenterol..

[59] Marrero J.A., Kulik L.M., Sirlin C.B., Zhu A.X., Finn R.S., Abecassis M.M., Roberts L.R., Heimbach J.K. (2018). Diagnosis, Staging, and Management of Hepatocellular Carcinoma: 2018 Practice Guidance by the American Association for the Study of Liver Diseases. Hepatology.

[60] Ayuso C., Rimola J., Vilana R., Burrel M., Darnell A., García-Criado Á., Bianchi L., Belmonte E., Caparroz C., Barrufet M. (2018). Diagnosis and staging of hepatocellular carcinoma (HCC): Current guidelines. Eur. J. Radiol..

[61] Omata M., Cheng A.-L., Kokudo N., Kudo M., Lee J.M., Jia J., Tateishi R., Han K.-H., Chawla Y.K., Shiina S. (2017). Asia-Pacific clinical practice guidelines on the management of hepatocellular carcinoma: A 2017 update. Hepatol. Int..

[62] Aghemo A. (2018). Update on HCC Management and Review of the New EASL Guidelines. Gastroenterol. Hepatol..

[63] de Lédinghen V., Vergniol J. (2008). Transient elastography (FibroScan). Gastroenterol. Clin. Biol..

[64] Mikolasevic I., Orlic L., Franjic N., Hauser G., Stimac D., Milic S. (2016). Transient elastography (FibroScan^®^) with controlled attenuation parameter in the assessment of liver steatosis and fibrosis in patients with nonalcoholic fatty liver disease—Where do we stand?. World J. Gastroenterol..

[65] Wong V.W.-S., Chan H.L.-Y. (2010). Transient elastography. J. Gastroenterol. Hepatol..

[66] Gennisson J.-L., Deffieux T., Fink M., Tanter M. (2013). Ultrasound elastography: Principles and techniques. Diagn. Interv. Imaging.

[67] Ferraioli G., Wong V.W.-S., Castera L., Berzigotti A., Sporea I., Dietrich C.F., Choi B.I., Wilson S.R., Kudo M., Barr R.G. (2018). Liver Ultrasound Elastography: An Update to the World Federation for Ultrasound in Medicine and Biology Guidelines and Recommendations. Ultrasound Med. Biol..

[68] Karlas T., Petroff D., Sasso M., Fan J.-G., Mi Y.-Q., De Lédinghen V., Kumar M., Lupsor-Platon M., Han K.-H., Cardoso A.C. (2018). Impact of controlled attenuation parameter on detecting fibrosis using liver stiffness measurement. Aliment. Pharmacol. Ther..

[69] Voican C.S., Louvet A., Trabut J.-B., Njiké-Nakseu M., Dharancy S., Sanchez A., Corouge M., Lamouri K., Lebrun A., Balian A. (2017). Transient elastography alone and in combination with FibroTest^®^ for the diagnosis of hepatic fibrosis in alcoholic liver disease. Liver Int..

[70] Anastasiou O.E., Büchter M., Baba H.A., Korth J., Canbay A., Gerken G., Kahraman A. (2016). Performance and Utility of Transient Elastography and Non-Invasive Markers of Liver Fiibrosis in Patients with Autoimmune Hepatitis: A Single Centre Experience. Hepat. Mon..

[71] Buechter M., Kahraman A., Manka P., Gerken G., Jochum C., Canbay A., Dechêne A. (2016). Spleen and Liver Stiffness Is Positively Correlated with the Risk of Esophageal Variceal Bleeding. Digestion.

[72] Buechter M., Manka P., Theysohn J.M., Reinboldt M., Canbay A., Kahraman A. (2018). Spleen stiffness is positively correlated with HVPG and decreases significantly after TIPS implantation. Dig. Liver Dis..

[73] Babu A.S., Wells M.L., Teytelboym O.M., Mackey J.E., Miller F.H., Yeh B.M., Ehman R.L., Venkatesh S.K. (2016). Elastography in Chronic Liver Disease: Modalities, Techniques, Limitations, and Future Directions. Radiographics.

[74] Ilan Y. (2007). Review article: The assessment of liver function using breath tests. Aliment. Pharmacol. Ther..

[75] Hsieh C., Yu C., Tzao C., Chu H., Chen T., Hsieh H., Liu Y., Yu J. (2006). Prediction of the risk of hepatic failure in patients with portal vein invasion hepatoma after hepatic resection. Eur. J. Surg. Oncol..

[76] Stehr A., Ploner F., Traeger K., Theisen M., Zuelke C., Radermacher P., Matejovic M. (2005). Plasma disappearance of indocyanine green: A marker for excretory liver function?. Intensive Care Med..

[77] Milazzo L., Piazza M., Sangaletti O., Gatti N., Cappelletti A., Adorni F., Antinori S., Galli M., Moroni M., Riva A. (2005). [13C]Methionine breath test: A novel method to detect antiretroviral drug-related mitochondrial toxicity. J. Antimicrob. Chemother..

[78] Afolabi P., Wright M., Wootton S.A., Jackson A.A. (2013). Clinical Utility of 13C-Liver-Function Breath Tests for Assessment of Hepatic Function. Dig. Dis. Sci..

[79] Miele L., Marrone G., Cefalo C., D’Achille S., Rapaccini G.L., Gasbarrini A., Grieco A. (2013). Potential use of liver function breath tests in the clinical practice. Eur. Rev. Med. Pharmacol. Sci..

[80] Oellerich M., Armstrong V.W. (2001). The MEGX Test: A Tool for the Real-Time Assessment of Hepatic Function. Ther. Drug Monit..

[81] Lorf T., Schnitzbauer A.A., Schaefers S.K.H., Scherer M.N., Schlitt H.J., Oellerich M., Becker H., Obed A. (2008). Prognostic value of the monoethylglycinexylidide (MEGX)-test prior to liver resection. Hepatogastroenterology.

[82] Fabris L., Jemmolo R.M., Toffolo G., Paleari D., Viaggi S., Rigon M., Casagrande F., Lirussi F., Strazzabosco M., Cobelli C. (1999). The monoethylglycinexylidide test for grading of liver cirrhosis. Aliment. Pharmacol. Ther..

[83] Stockmann M., Lock J.F., Malinowski M., Niehues S.M., Seehofer D., Neuhaus P. (2010). The LiMAx test: A new liver function test for predicting postoperative outcome in liver surgery. HPB.

[84] Stockmann M., Lock J.F., Riecke B., Heyne K., Martus P., Fricke M., Lehmann S., Niehues S.M., Schwabe M., Lemke A.-J. (2009). Prediction of Postoperative Outcome After Hepatectomy With a New Bedside Test for Maximal Liver Function Capacity. Ann. Surg..

[85] Buechter M., Thimm J., Baba H.A., Bertram S., Willuweit K., Gerken G., Kahraman A. (2019). Liver Maximum Capacity: A Novel Test to Accurately Diagnose Different Stages of Liver Fibrosis. Digestion.

[86] Buechter M., Kersting S., Gerken G., Kahraman A. (2019). Enzymatic liver function measured by LiMAx—A reliable diagnostic and prognostic tool in chronic liver disease. Sci. Rep..

[87] Buechter M., Gerken G., Hoyer D.P., Bertram S., Theysohn J.M., Thodou V., Kahraman A. (2018). Liver maximum capacity (LiMAx) test as a helpful prognostic tool in acute liver failure with sepsis: A case report. BMC Anesthesiol..

[88] Kreimeyer H., Buechter M., Best J., Gieseler R.K., Katsounas A., Sowa J.-P., Gerken G., Canbay A., Manka P., Bechmann L.P. (2022). Performance of the Liver Maximum Function Capacity Test, Fibrinogen, and Transient Elastography in Patients with Acute Liver Injury. Dig. Dis..

[89] Lock J.F., Kotobi A.N., Malinowski M., Schulz A., Jara M., Neuhaus P., Stockmann M. (2013). Predicting the prognosis in acute liver failure: Results from a retrospective pilot study using the LiMAx test. Ann. Hepatol..

[90] Jara M., Malinowski M., Lüttgert K., Schott E., Neuhaus P., Stockmann M. (2015). Prognostic value of enzymatic liver function for the estimation of short-term survival of liver transplant candidates: A prospective study with the LiMAx test. Transpl. Int..

[91] Schurink I.J., de Haan J.E., Willemse J., Mueller M., Doukas M., Roest H., de Goeij F.H.C., Polak W.G., Ijzermans J.N.M., Dutkowski P. (2021). A proof of concept study on real-time LiMAx CYP1A2 liver function assessment of donor grafts during normothermic machine perfusion. Sci. Rep..

[92] Anger F., Klein I., Löb S., Wiegering A., Singh G., Sperl D., Götze O., Geier A., Lock J.F. (2021). Preoperative Liver Function Guiding HCC Resection in Normal and Cirrhotic Liver. Visc. Med..

[93] Rahimli M., Perrakis A., Gumbs A.A., Andric M., Al-Madhi S., Arend J., Croner R.S. (2022). The LiMAx Test as Selection Criteria in Minimally Invasive Liver Surgery. J. Clin. Med..

[94] Heucke N., Wuensch T., Mohr J., Kaffarnik M., Arsenic R., Sinn B., Müller T., Pratschke J., Stockmann M., Sack I. (2019). Non-invasive structure–function assessment of the liver by 2D time-harmonic elastography and the dynamic Liver MAximum capacity (LiMAx) test. J. Gastroenterol. Hepatol..

[95] Schmitz S.M.-T., Kroh A., Koch A., Brozat J.F., Stier C., Neumann U.P., Ulmer T.F., Alizai P.H. (2021). Comparison of Liver Recovery after Sleeve Gastrectomy and Roux-en-Y-Gastric Bypass. Obes. Surg..

[96] Blüthner E., Jara M., Shrestha R., Faber W., Pratschke J., Stockmann M., Malinowski M. (2019). The predictive value of future liver remnant function after liver resection for HCC in noncirrhotic and cirrhotic patients. HPB.

[97] Rashidi-Alavijeh J., Kahraman A., Gerken G., Theysohn J.M., Willuweit K., Hoyer D.P., Lange C.M., Buechter M. (2021). Enzymatic liver function measured by LiMAx is superior to current standard methods in predicting transplant-free survival after TIPS implantation. Sci. Rep..

[98] Reichert M.C., Schulz A., Massmann A., Buecker A., Glanemann M., Lammert F., Malinowski M. (2020). Predictive Power of Liver Maximum Function Capacity Test in Transjugular Intrahepatic Portosystemic Shunt Patients: A Pilot Study. Dig. Dis..

[99] Reichert M.C., Massmann A., Schulz A., Buecker A., Glanemann M., Lammert F., Malinowski M. (2021). Volume–Function Analysis (LiMAx Test) in Patients with HCC and Cirrhosis Undergoing TACE—A Feasibility Study. Dig. Dis. Sci..

[100] Barzakova E.S., Schulze-Hagen M., Zimmermann M., Lurje G., Bednarsch J., Pedersoli F., Isfort P., Kuhl C., Bruners P. (2019). Monitoring Liver Function of Patients Undergoing Transarterial Chemoembolization (TACE) by a 13C Breath Test (LiMAx). Cardiovasc. Interv. Radiol..

[101] Senk K., Wilcke J., Haimerl M., Verloh N., Bartulos C.R., Bäumler W., Stroszczynski C., Wiggermann P. (2021). Prediction of transarterial chemoembolization (TACE) outcome by pre- and postinterventional 13C-methacetin breath test. Clin. Hemorheol. Microcirc..

[102] Parmar K.L., O’Reilly D., Valle J.W., Braun M., Naish J.H., Williams S.R., Lloyd W.K., Malcomson L., Cresswell K., Bamford C. (2020). Prospective study of change in liver function and fat in patients with colorectal liver metastases undergoing preoperative chemotherapy: Protocol for the CLiFF Study. BMJ Open.

[103] Jara M., Bednarsch J., Malinowski M., Pratschke J., Stockmann M. (2016). Effects of oxaliplatin-based chemotherapy on liver function—An analysis of impact and functional recovery using the LiMAx test. Langenbeck’s Arch. Surg..

[104] Bednarsch J., Jara M., Lock J.F., Malinowski M., Pratschke J., Stockmann M. (2015). Noninvasive diagnosis of chemotherapy induced liver injury by LiMAx test—Two case reports and a review of the literature. BMC Res. Notes.

[105] Lock J.F., Westphal T., Rubin T., Malinowski M., Schulz A., Jara M., Bednarsch J., Stockmann M. (2017). LiMAx Test Improves Diagnosis of Chemotherapy-Associated Liver Injury Before Resection of Colorectal Liver Metastases. Ann. Surg. Oncol..

[106] Ahn J.C., Connell A., Simonetto D.A., Hughes C., Shah V.H. (2020). Application of Artificial Intelligence for the Diagnosis and Treatment of Liver Diseases. Hepatology.

[107] Lee H.W., Sung J.J.Y., Ahn S.H. (2021). Artificial intelligence in liver disease. J. Gastroenterol. Hepatol..

[108] Nam D., Chapiro J., Paradis V., Seraphin T.P., Kather J.N. (2022). Artificial intelligence in liver diseases: Improving diagnostics, prognostics and response prediction. JHEP Rep..

